# Case Report: Disseminated Systemic Embolism of Lipiodol After Lymphography for Plastic Bronchitis After Fontan Repair

**DOI:** 10.3389/fped.2020.584185

**Published:** 2020-10-27

**Authors:** Jelena Hubrechts, Håkan Wåhlander, Cecilia Kjellberg-Olofsson, Geert Maleux, Marc Gewillig

**Affiliations:** ^1^Department of Pediatric and Congenital Cardiology, Leuven University Hospital, Leuven, Belgium; ^2^Pediatric Heart Center, The Queen Silvia Children's Hospital, Gothenburg, Sweden; ^3^Department of Pediatrics, Institute for Clinical Sciences, Sahlgrenska Academy, Gothenburg University, Gothenburg, Sweden; ^4^Department of Pediatrics, Sundsvall Hospital, Sundsvall, Sweden; ^5^Interventional Radiology, Leuven University Hospital, Leuven, Belgium

**Keywords:** plastic bronchitis, lymphography, lymphangiography, lipiodol, cerebral embolism

## Abstract

Lipiodol-based lymphangiography is not only a diagnostic tool for visualization of lymphatic disorders such as plastic bronchitis (PB), but also aims a therapeutic effect by embolizing lymph leakages. We performed such percutaneous lymphatic embolization for PB in a Fontan patient with proven absence of right-to-left shunt, and demonstrated important lymphatic abnormalities in the mediastinum. Shortly after the procedure, the patient developed severe convulsive seizures, revealing multiple cerebral embolisms of Lipiodol. Radiological images were impressive, yet the clinical neurological outcome was favorable. Lipiodol-based lymphography in Fontan patients with plastic bronchitis should be avoided as this subgroup is more likely to have developed lympho-pulmonary venous connections which allow systemic emboli.

## Introduction

Plastic bronchitis (PB) is a rare but severe complication in patients with Fontan-circulation (1, 2). Pathophysiology of lymphatic flow in this type of patients is still insufficiently understood. Dori et al. showed that most such patients have abnormal centrifugal flow from the mediastinal lymphatics into the airways with secondary formation of bronchial casts (3). These findings have revived the interest and application of lymphatic investigations and therapies.

Dynamic lymphangiography gives morphologic and functional information and is essential to understand why lymphatics fail in a specific patient in order to assess lymphatic treatment options. Currently two contrast agents are available in clinical practice: ethiodized oil (Lipiodol®, Aulnay-sous-Bois, Guerbet, France) which allows visualization with conventional radiologic equipment, and gadolinium, a heavy ferromagnetic metal which requires magnetic resonance imaging.

Lipiodol is a water insoluble contrast agent, a naturally iodinated fatty acid ethyl ester of poppy seed oil, developed in 1901; over the years it has been used, amongst others, in lymphangio-, myelo- and hysterosalpingography and certainly chemoembolization. Until the 1980's when CT took over, it was routinely used to assess metastases within the lymphatic system. Currently it is still used in lymphangiography and in embolization procedures such as post-operative lymphatic leakages. Lipiodol remains for several hours and days in the lymphatic system, allowing localization of downstream nodes; this feature is used in recent techniques such as direct transabdominal puncture of the cysterna chyli for transabdominal access to the thoracic duct (4). An additional property is its sclerosing effect at the leakage site where lymph leaves the lymphatic vessels (5); as such it has been used therapeutically with variable effect in case of chylothorax, chyloperitoneum, protein losing enteropathy, plastic bronchitis, and other lymphatic diseases. However, when Lipiodol enters the bloodstream, it will be carried as small oily droplets until the next capillary circuit, typically the lungs, where it may cause temporary micro-occlusions. Lipiodol is therefore absolutely contra-indicated in patients with a right-to-left shunt as this may cause systemic embolization to vital organs.

Recently Dori and coworkers successfully treated PB with targeted embolization of abnormal lymphatic vessels originating from the thoracic duct with centrifugal flow into the mediastinum (3, 6, 7). They recommended inguinal lymphangiography with Lipiodol not only to identify the lymphatic thoracic problem, but also to localize the cysterna chyli, thereby allowing its direct puncture for access to the thoracic duct. However, in a series of 18 patients with plastic bronchitis they observed “paradoxical” cerebral embolization in one patient even in the absence of a venous right-to-left shunt (3, 8).

We describe a similar case of a pediatric patient with plastic bronchitis after Fontan-palliation, who underwent percutaneous lymphatic embolization with Lipiodol and developed multi-organ failure due to systemic Lipiodol embolisms.

## Case Report

A 12-year-old boy with trisomy 21 and unbalanced atrio-ventricular septum defect, borderline left heart structures and coarctation of the aorta received an extracardiac total cavopulmonary connection at the age of 5 years. He developed plastic bronchitis 4 years later. A catheterization showed a pulmonary artery pressure of 13 mmHg, transpulmonary gradient of 5 mmHg, cardiac index of 1.8 L/min/m^2^ and pulmonary vascular resistance of 3.4 WU × m^2^. A significant intracardiac right-to-left shunt was excluded by the absence of arterial desaturation, by cardiac catheterization with high volume fast contrast injection in the caval veins in subtraction mode, confirming the absence of residual fenestration and venovenous collaterals; arteriovenous malformations in the lungs were excluded by cardiac catheterization and thoracic CT scan. In the months prior to the procedure he was hospitalized on several occasions for 10 days or longer due to exacerbations of PB with expectoration of casts and hypoxia. His medication consisted of bosentan, sildenafil, acetylsalicylic acid, furosemide, spironolactone, levothyroxine, montelukast, prednisone, inhalations of salbutamol and steroids, and at relapses inhalations of t-PA.

**Figure 1:**
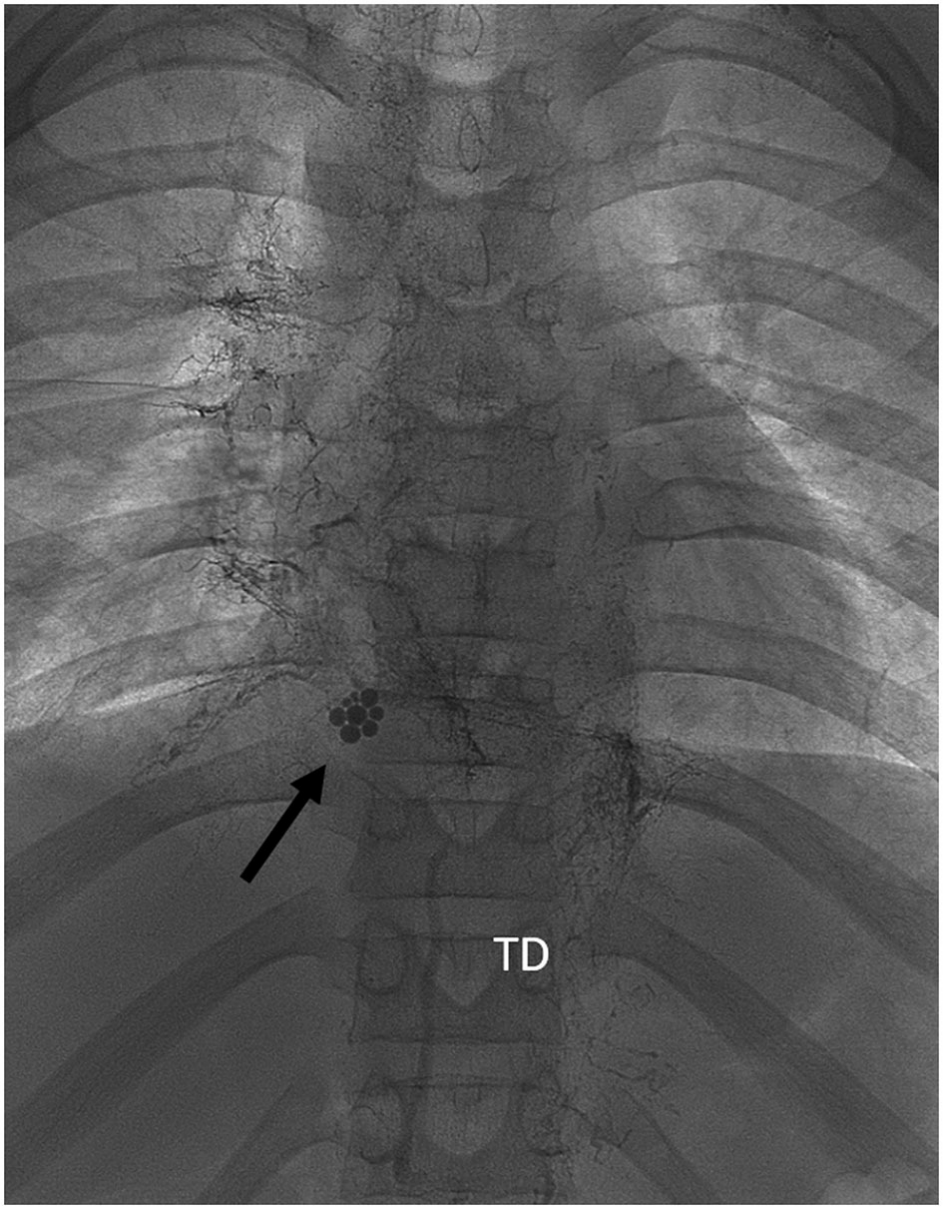
Frontal view of fluoroscopic images during inguinal injection of Lipiodol. At abdominal level there is opacification of lymph vessels with drainage of Lipiodol into the cysterna chyli and thoracic duct (TD). Image of occlusion of the TD on the passage from abdominal to thoracic level. Black arrow: droplets of ethiodized oil at the passage from the inferior caval vein to the TCPC conduit, revealing early lymphovenous shunting. At thoracic level there is drainage of Lipiodol through multiple collaterals to hilar, peribronchial, mediastinal, and axillar dilated lymph vessels. Right mediastinum is more affected than the left.

**Figure 2:**
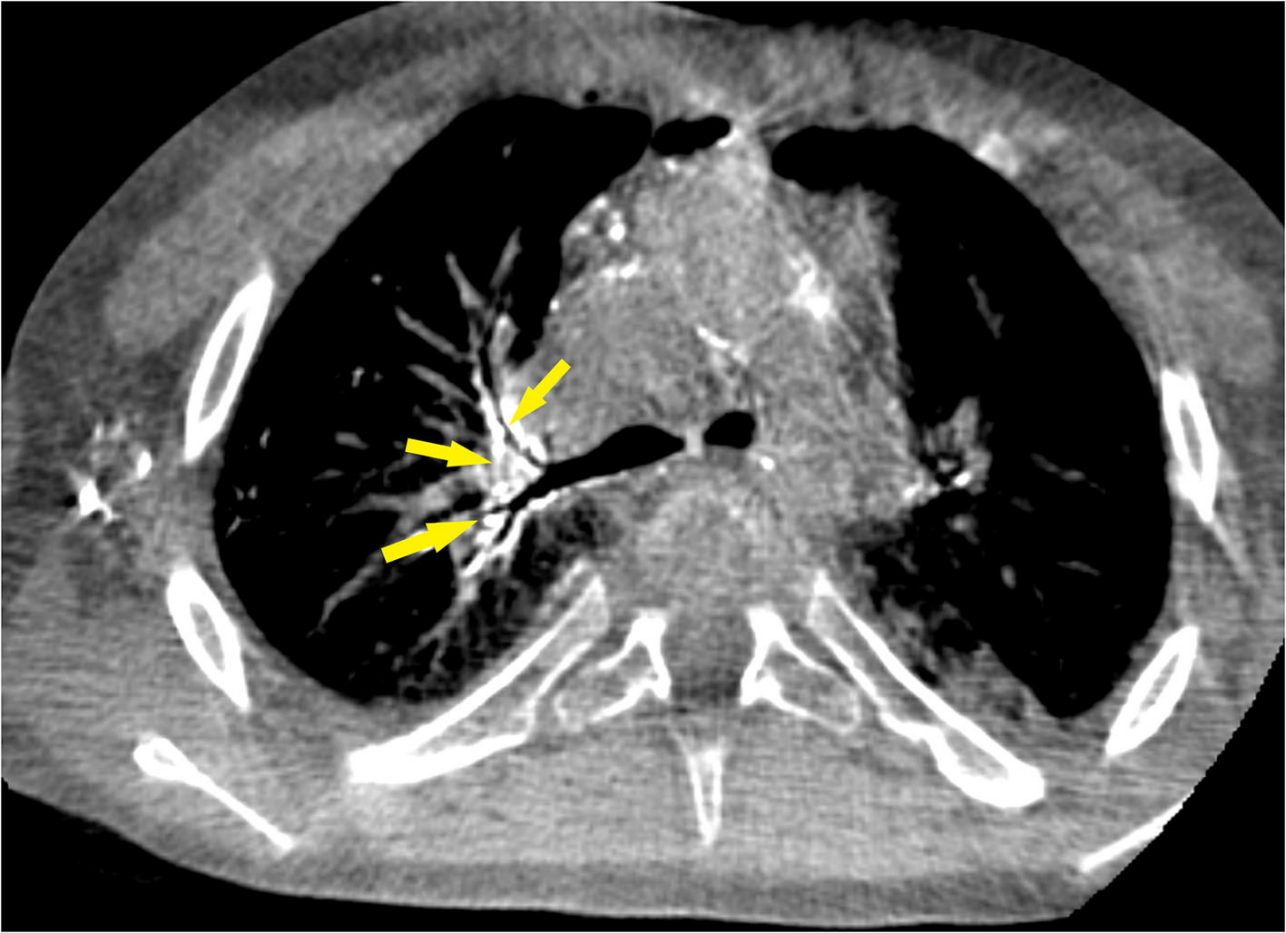
Cone-beam computed tomography image during Lipiodol-based lymphangiography shows filling of several mediastinal abnormal dilated lymph vessels, predominantly peribronchial right (yellow arrows). This is similar to the fluoroscopic image of Figure 1.

He was admitted to our hospital for lymphatic embolization; as part of the work-up and possible treatment an inguinal Lipiodol-based lymphangiography was scheduled. Informed consent was obtained from the patient and his parents. The procedure was performed under general anesthesia. The lymph nodes in both groins were visualized using a linear 12 MHz ultrasound transducer. A suitable inguinal node was identified and directly punctured with a 22G spinal needle (BD Spinal Needle, Guadalix, Madrid, Spain). Ethiodized oil (Lipiodol® Ultrafluid®, Guerbet, Aulnay-sous-Bois, France) was slowly injected under fluoroscopic guidance until opacification of the efferent lymph vessels of the lymph node was observed. The progression of Lipiodol through the iliac and retroperitoneal lymph vessels, the cisterna chyli and the beginning of the TD was observed under fluoroscopic guidance (Figure 1); there was no evidence of a TD at thoracic level, excluding direct targeting of the thoracic duct. After the initial filling phase, injection was continued until a clear lymphatic leak into the mediastinum was visualized and well-filled with Lipiodol. A total of 18 ml of Lipiodol was injected. The dose limit recommended by the manufacturer (8 ml per limb) was not taken into account. After the procedure, a cone beam CT scan of the thoracic base was performed to evaluate the lymphatic anatomy. Multiple lymphatic collaterals that drained to hilar, peribronchial, mediastinal and axillar regions were demonstrated (Figure 2).

**Figure 3:**
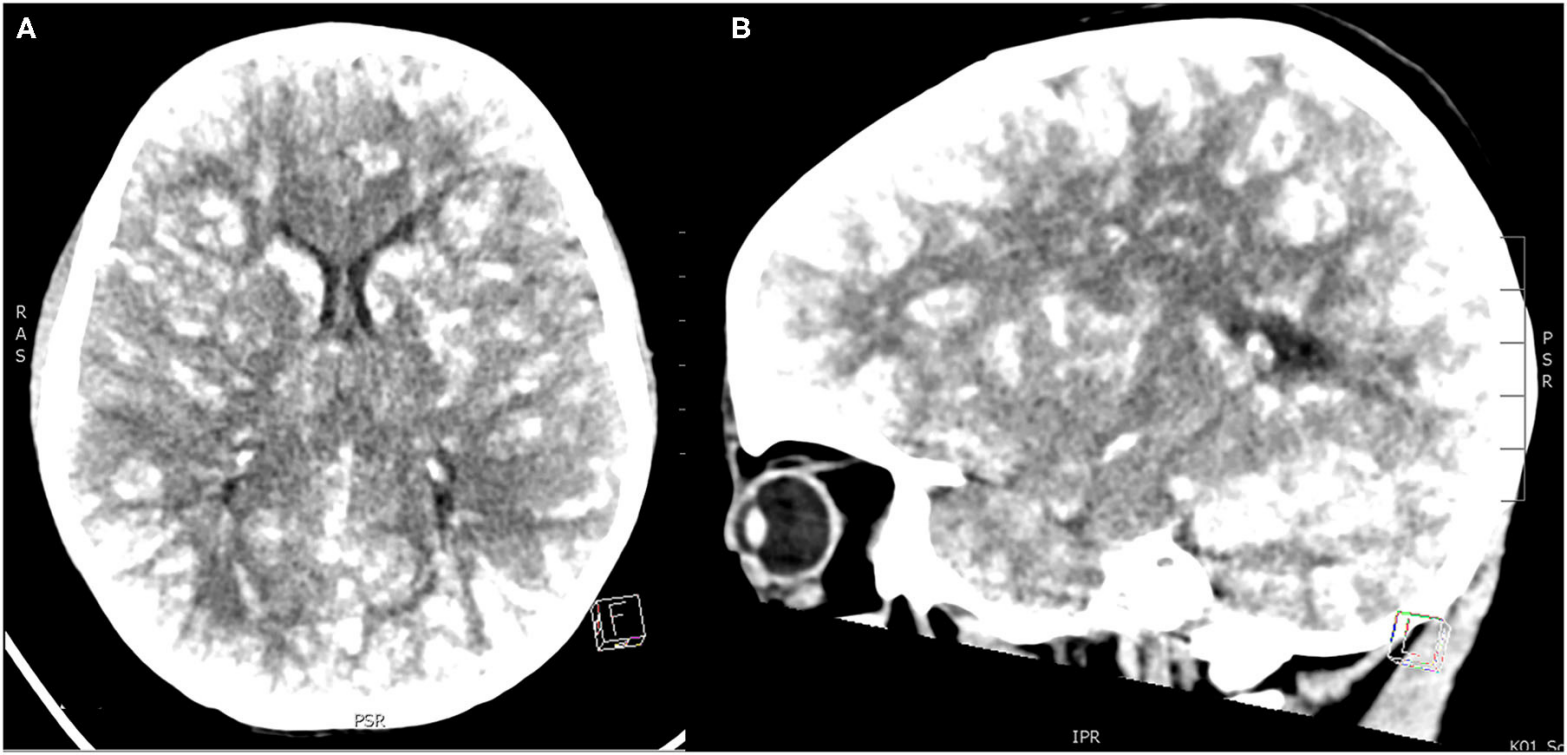
**(A,B)** Unenhanced head CT images with “lâcher de ballons.” Lipiodol is shown as diffuse high-density lesions in cortical, subcortical region and more profound in the white substance and in cerebral and cerebellar gray cores. (**A)** axial, (**B)** sagittal section.

Three hours after procedure, the patient developed severe convulsive seizures with loss of consciousness. He was intubated and sedated, levetiracetam was started. Urgent cerebral computed tomography showed diffuse, supra- and infratentorial, bilateral hyperdensities of more than 90 Hounsfield units, diagnostic for Lipiodol emboli. Other cerebral insults such as different types of stroke were excluded by the very disseminated topography of the lesions (Figure 3).

**Figure 4:**
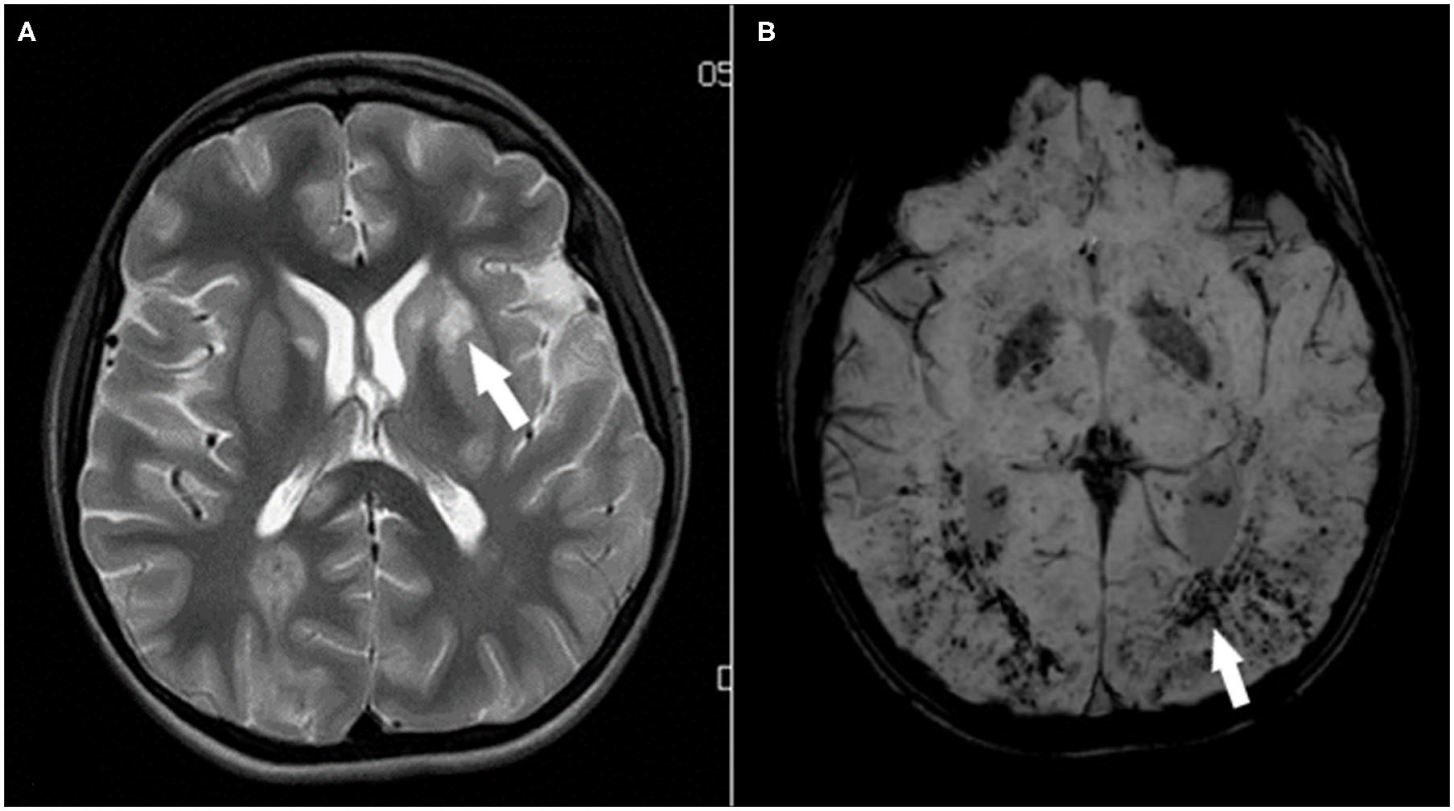
**(A,B)** Cerebral MRI 14 days after Lipiodol embolism. (**A)** Axial T2-weighted magnetic resonance imaging of the basal ganglia, showed diffuse high signal lesions, for example in the left putamen (arrow). Also, periventricular and subcortical white matter hyperintensities in both hemispheres, corresponding to edema after recent ischemia. (**B)** Susceptibility-weighted imaging (SWI) highlighted the presence of hemorrhagic components within the infarcted zones and diffuse, countless residual (micro-) hemorrhages (arrow) in supra- and infratentorial cerebral parenchyma.

The patient developed multiple organ failure. Neurologically, he was non-responsive. Hemodynamically, inotropics, and vasopressors were required to maintain systemic perfusion. He developed acute kidney failure with oliguria 0.3 ml/kg/h and creatinemia of 1.3 mg/dl, and liver dysfunction with cytolysis (transaminases > 2.000 U/L). Radiological work-up with thoracic and abdominal CT showed diffuse deposits of Lipiodol in lungs, heart, spleen, kidneys and in less amount, thyroid, liver, stomach and intestines. Cerebral CT was repeated 48 h later, with still impressive images of cerebral emboli, but yet less hyperdense. He was sedated and intubated for a total of 15 days. Liver enzymes as renal function recovered to normal levels. Cerebral MRI was performed 14 days later and confirmed multiple diffuse bilateral lesions of the cerebral parenchyma, yet less extended than on the previous CT (Figure 4). On T2 weighted images, high signal lesions, mainly in the frontoparietal cortex, cerebral and cerebellar gray cores and in periventricular and subcortical white substance, corresponded to vasogenic and cytotoxic edema after recent ischemia caused by Lipiodol emboli. Susceptibility weighted imaging SWI, very sensitive for blood residues, was added to the standard MRI sequences and highlighted the presence of hemorrhagic components within the infarcted zones. Disseminated hemosiderin deposits reflected diffuse residual (micro-)hemorrhages in supra- and infratentorial cerebral parenchyma as in the brainstem. Additionally, he developed a recurrent left and right chylothorax which necessitated pleural drainage for 3 weeks.

The patient could progressively be weaned from all sedatives (diazepam, midazolam, clonidine). Neurologically, there were involuntary dyskinetic hand and arm movements and a language deficit. Six months after the procedure his neurological status has almost recovered, except for development of spasticity in the lower extremities and persistent mental fatigue. All other organ functions had normalized after 2 months.

During the first 3 months after the lymphatic intervention, the patient was virtually free from bronchial casts. However, during the next 6 months, he began having relapse of PB with bronchial casts, which gradually progressed in frequency to a situation similar to the situation before the lymphatic intervention. Eleven months after the procedure, he is still on prednisone and intermittent t-PA inhalations according to the protocol which was used before the procedure.

## Discussion

Systemic embolism of Lipiodol is a rare but severe and possibly lethal complication. In retrospect, as significant right-to-left shunts were excluded during the pre-procedural work-up, the massive systemic emboli suggest abnormal connections between the lymphatic system and the pulmonary veins or directly to the atrium. The thoracic cone-beam CT showed retrograde lymph flow into the atrial wall, with possible leak. We speculate that the systemic emboli were caused by abnormal lympho-pulmonary connections.

Lymphatic systemic embolization of Lipiodol is very rare in Fontan patients in general despite the obligatory pressure difference between systemic and pulmonary veins. Systemic embolism is more likely to occur in the subgroup of Fontan patients with plastic bronchitis, but not in those with protein losing enteropathy. Indeed, such patients exhibit frequent lymphatic abnormalities even before Fontan completion (9). Very frequently Fontan patients with plastic bronchitis have an additional problem of the thoracic duct (obstruction, occlusion, absence). The TD is a large vessel connecting the abdominal cysterna chyli to the left subclavian vein. However, the TD is not a simple pathway as suggested by its name, but rather a multivalved contractile vessel functioning like a pump, allowing to propel fluid at a differential pressure as high as 40–70 mmHg (10, 11). The junction of the TD with the subclavian vein is guarded by a valve, but in chronic venous congestion as in Fontan patients, blood may enter the dilated TD and coagulate within the lymphatic vessel. If the normal passage to the subclavian vein is obstructed or ill-directed, this powerful pump can “blow” its content into the mediastinum and neck region. In the absence of a TD, lymphangions and smaller lymphatic vessels will connect to neighboring (systemic or apparently pulmonary) veins and pump the lymph into that vessel. However, overdistended submucosal lymphatics can tear and leak allowing lymph to flow into the bronchial space, causing the typical casts of PB.

Specific to the procedure, we aimed to both visualize and treat the lymphatic leak and therefore fully saturated the lymphatic system with Lipiodol. Once the Lipiodol is injected into the lymphatic system, there is no possibility to retrieve the substance, nor to dilute the concentration. To our knowledge, it is impossible to exclude in advance abnormal lymphatic connections to the pulmonary veins. Such minute and low-flow connections cannot be ruled out in advance; their existence can be presumed when in the absence of a significant venous right-to-left shunt systemic emboli appear massively minutes-hours after intralymphatic injection; avoiding or minimizing the amount of oily contrast agent used is of utmost importance. In our experience with two patients who underwent inguinal Lipiodol lymphography for plastic bronchitis, one developed systemic embolism. The other patient remains cast-free now with 20 months of follow-up. In the series of Philadelphia, 1 patient in 18 had cerebral Lipiodol embolism (3, 6). He fully recovered 1 month after procedure. Despite the disastrous pattern in the radiological images, our patient also had a good neurological outcome. Lipiodol is known for its typical high radiopacity on (neuro-) imaging but apparently, is less toxic in organic tissue. The uncountable number of micro-hemorraghes on MRI (Figure 4) suggest Lipiodol occlusion of small arteries; similar images are seen in critical ill patients with hypoxia, which possibly accentuated the phenomenon of microbleedings. The regression of clinical symptoms and resorption of the emboli from the first CT to the second and later on MRI is remarkable.

Since this case the investigation and treatment protocol of PB in our center was adapted. For anatomical and functional visualization of the lymphatic system, inguinal intranodal Gadolinium Dynamic Contrast-enhanced Magnetic Resonance lymphangiography (DCMRL) is performed. Adequate visualization is obtained; however, DCMRL does not permit direct treatment of the dilated leaking lymph vessels, contrary to the sclerosing effect of Lipiodol-based procedures. Because of its low radiodensity and fast progression, DCMRL does not allow easy fluoroscopic localization of the cysterna chyli for accessing the TD, which makes intervention on mediastinal lymphatics via transductal access very difficult. Nevertheless, some alternative techniques to treat PB after Fontan repair exist. Lymphatic leaks in PB can also be sealed without transductal access by direct peritracheal puncture techniques either percutaneous or transtracheal (12, 13).

## Conclusion

Cerebral embolism of Lipiodol after lymphography is a very severe complication in Fontan- patients with PB. We presume the existence of abnormal lymphatic connections to the pulmonary veins which are not possible to exclude in advance. When in doubt in high risk patients such as those with PB after Fontan, avoiding or minimizing the amount of oily contrast agent used is indicated. Other techniques need to be explored.

## Data Availability Statement

The raw data supporting the conclusions of this article will be made available by the authors, without undue reservation.

## Ethics Statement

Ethical review and approval was not required for the study on human participants in accordance with the local legislation and institutional requirements. Written informed consent to participate in this study was provided by the participants' legal guardian/next of kin. Written informed consent was obtained from the minor(s)' legal guardian/next of kin for the publication of any potentially identifiable images or data included in this article.

## Author Contributions

MG and GM conceptualized the design of the study and reviewed and revised the manuscript. JH drafted the initial manuscript. HW and CK-O referred the patient, provided follow-up, and long-term data. All authors contributed to manuscript revision, read, and approved the submitted version.

## Conflict of Interest

The authors declare that the research was conducted in the absence of any commercial or financial relationships that could be construed as a potential conflict of interest.
